# The Challenges of Including Patients With Aphasia in Qualitative Research for Health Service Redesign: Qualitative Interview Study

**DOI:** 10.2196/12336

**Published:** 2020-02-07

**Authors:** Sarah Prior, Andrea Miller, Steven Campbell, Karen Linegar, Gregory Peterson

**Affiliations:** 1 School of Medicine University of Tasmania Burnie Australia; 2 School of Health Sciences University of Tasmania Launceston Australia; 3 Tasmanian Health Service Burnie Australia; 4 College of Health and Medicine School of Pharmacy University of Tasmania Hobart Australia

**Keywords:** stroke, communication, research, qualitative, aphasia, participatory research

## Abstract

**Background:**

Aphasia is an impairment of language, affecting the production or comprehension of speech and the ability to read or write. Aphasia is a frequent complication of stroke and is a major disability for patients and their families. The provision of services for stroke patients differs across health care providers and regions, and strategies directed at improving these services have benefited from the involvement of patients. However, patients with aphasia are often excluded from these co-design activities due to a diminished capacity to communicate verbally and a lack of health researcher experience in working with patients with aphasia.

**Objective:**

The primary aim of this paper is to identify approaches appropriate for working with patients with aphasia in an interview situation and, more generally, determine the importance of including people with aphasia in health service improvement research. The secondary aim is to describe the experiences of researchers involved in interviewing patients with aphasia.

**Methods:**

A total of 5 poststroke patients with aphasia participated in face-to-face interviews in their homes to gain insight into their in-hospital experience following their stroke. Interviews were audio-recorded, and thematic analysis was performed. The experiences of the researchers interviewing these patients were informally recorded postinterview, and themes were derived from these reflections.

**Results:**

The interview technique utilized in this study was unsuitable to gain rich, qualitative data from patients with aphasia. The experience of researchers performing these interviews suggests that preparation, emotion, and understanding were three of the main factors influencing their ability to gather useful experiential information from patients with aphasia. Patients with aphasia are valuable contributors to qualitative health services research, and researchers need to be flexible and adaptable in their methods of engagement.

**Conclusions:**

Including patients with aphasia in health service redesign research requires the use of nontraditional interview techniques. Researchers intending to engage patients with aphasia must devise appropriate strategies and methods to maximize the contributions and valuable communications of these participants.

## Introduction

### What is Known?

Patient involvement in health care service redesign is recognized as an important component of ensuring quality, evidence-based care. Termed co-led redesign, this method has been frequently utilized to develop and deliver improved health care services for a variety of patient groups. For instance, successful outcomes from co-design have been shown in chronic illnesses [1] where patients were involved in developing technological solutions for mobilizing personal strengths, in the provision of youth mental health services [2], and in developing stroke rehabilitation priorities [3].

Stroke is the leading cause of aphasia [4], a disorder of communication, and Australian data suggests that approximately 29% of patients with acute stroke present to hospital with aphasia [5]. About 40% of all people who experience a stroke develop aphasia, with variation in the severity ranging from mild cases, with occasional difficulties in word-finding, to total loss of oral output [6]. It is one of the most devastating symptoms in stroke survivors and severely affects patients’ communication, quality of life, and social interactions [6].

### What is the Gap?

Although a number of methods have previously been described for involving patients with aphasia in qualitative research [7-10], there is little information in the literature describing why it is important to include patients with aphasia in co-design research or the experiences of the research team members who are involved in obtaining meaningful data from patients with aphasia. The primary aim of qualitative research is to understand the experience from the perspective of the participant. However, the researchers, either consciously or unconsciously, bring to the research setting their predispositions, assumptions, and beliefs, which may or may not be beneficial [11]. In this case, face-to-face interviews require careful thought and consideration of the type of aphasia the patient has, how best to communicate with the patient, and how the patient is most easily able to communicate with the interviewer, which is something that an inexperienced interviewer may find confronting and challenging.

### What is the Purpose?

This study aims to highlight the need for the use of appropriate methods to interview people with aphasia as part of a co-design approach to health service improvement, based on the experiences of researchers who interviewed stroke patients with aphasia. This paper also discusses what was learned from the experiential accounts of patients with post-stroke aphasia regarding health service improvement. This study fills a gap in current knowledge around the importance of including individuals with aphasia in broad qualitative research that is not specifically targeting people with communication difficulties, and how the experiences of researchers may affect the outcomes of this research.

## Methods

### Recruitment

Overall, 117 poststroke patients from a rural health district were invited to participate in face-to-face interviews as part of a local, co-design, health service improvement initiative. A total of 27 (23%) patients accepted this invitation. Of these patients, 5 (19%) had some form of communication difficulty (aphasia), which was not known to the researchers in four cases before conducting the interviews.

### Procedure

The interviews were held in a place of the patient’s choosing. In total, 22 interviews were held in the patient’s home, 3 over the phone (interstate residents), and the remaining 2 in a public café. All interviews with patients with aphasia took place in the home of the patient, with their spouses, family members, or carers present as required or requested. Each interview consisted of a set of semistructured questions, with time allowing for the conversation to flow in the direction set by the participant and their family or carers. Interviews with participants with aphasia ranged in time from 47 minutes to 1 hour and 50 minutes, which was consistent with interviews with participants without aphasia.

### Analysis

All interviews were audio-recorded, and this data was later transcribed for thematic analysis, which will not be presented in this paper. The researchers who were involved in interviewing the participants with aphasia reflected on their experiences through conversations with the research team, and reflections were documented and reviewed. This paper will present the reflections of the researchers as major themes from the data.

## Results

### Researcher Technique and Experience

The results suggest that there were three major factors/themes that influenced researcher experience when interviewing patients with aphasia: preparation, emotion, and understanding.

### Preparation

Having not known, or inquired, in advance about any potential communication difficulties for the poststroke patients, the researchers conducting interviews with individuals with aphasia felt unprepared in several ways. Firstly, they felt that they were ineffective at communicating with the participant and their families/carers:

I felt as though I was never looking at the right person or asking the right questions.

Researcher 1

Secondly, the ability to record the nonverbal communication was hindered as the research plan only included an audio recording of the interview. In this instance, researchers made written notes after the interview based on what was recalled about the nonverbal communication.

I found myself sitting on a public park bench scribbling as much as I could remember about the way he had interacted with me non-verbally.

Researcher 2

### Emotion

When it became apparent that the participant had aphasia, this elicited a range of emotions within the researchers. These included relief that there was a family member (or carer) present, anxiety about being able to effectively communicate, embarrassment at not being well-prepared, and fear of being judged.

I was shocked to see that the patient was having difficulty speaking but was very relieved when his partner was able to fill in the gaps for me.

Researcher 3

### Understanding

It was important for the research team to ensure that the information they collected during the interviews was a correct reflection of what the participant wanted to share. At times, it was physically difficult to understand the conversation and its context. It was also difficult for the researcher to develop an understanding of the patient experience due to the challenges faced because of the communication deficits.

I could interpret some of what he was saying but it was hard for me to gain an understanding of where it fit in his care or his experience.

Researcher 1

### Patient Experience

The interview data collected from the five patients with aphasia indicated a perspective of poststroke care which differed from those who did not have communication deficits. Themes highlighted more often by participants with aphasia were the need for a more focused approach to poststroke communication development, family involvement, and ongoing education. The full results and thematic analysis will be published at a later date.

It was noted that during transcription there were gaps in the interpretation of the material collected during the interviews with patients with aphasia. These gaps could have been avoided through the use of different methods of recording, either by recording video as well or by having a second interviewer involved in note-taking. It was noted that there was a disconnect between the experience within the interview and the recording that was translated. After spending some time with the participants a clear method of communicating was often established, though it was not always verbal. For example, confirming with a participant what it is that they were saying often resulted in nodding, clapping of hands, or using another form of movement to respond. This exchange was lost in the recording and the translation and was difficult to understand without having been personally involved in the interview. Other methods of communication included patting the interviewer’s hand or leg when wanting to speak, showing excited facial expressions when confirming a story or comment, and squeezing the hand or the leg of the interviewer when having something to say.

## Discussion

### Primary Findings

This study aimed to provide an improved understanding of working with patients with aphasia in a qualitative research context. We have described the approach used in our study to interview poststroke patients with communication difficulties to highlight the weaknesses of not having a good prior understanding of working with this patient group. Audio recording as a blanket means of data collection is inadequate for interviewing patients with aphasia, and utilizing methods such as video recording [7,8] or amending the question material [9] would have been beneficial in strengthening this research.

Before the interviews took place, participants with aphasia did not necessarily disclose their communication difficulties or the extent to which they relied on nonverbal communication. This created a range of complexities for the research team, most notably that they were underprepared. At times, the interviewers questioned their techniques, as they found themselves assisting participants by finishing sentences, offering words to complete stories, or prompting the patient about what they were meaning to say. Again, despite having much experience with face-to-face interviews, working with participants with communication difficulties can be challenging and confronting at times, creating a sense of anxiety, frustration, and embarrassment. Having family members present in some of the interviews helped not only in terms of communication, but also to jog the memory of the patient about their experience in the hospital. It was noted that participants who had difficulty communicating verbally often relied on their family members to speak for them, consistent with previous findings [10]. This approach assisted the researcher in gathering experiential information, but the context of some conversations remained unclear.

It was also noted that there seemed to be no frustration among the participants, their spouses, or family members during the interviews, which was a surprising and pleasing finding given previous research about the difficulties faced by patients with aphasia and their families [12]. This process and the perceptions of family members confirm that patients with communication difficulties were grateful to have their experiences heard, and they were happy to provide their opinions and feedback on the health care services they received.

Interviewing participants as part of qualitative research has been nominated as a gold standard method over many years, with audio recording considered a sound choice. However, working with participants who have communication deficits shows that verbal interviews often lack content, including emotion, which can be an important factor in discussing experiences. In addition to the barrier present in communicating with the participant with aphasia, there is difficulty in judging thir response, particularly if a voice recorder is the only method utilized. Based on our results, in a co-design study, it would be beneficial to consult with the patient or their family/carer before the interview to ascertain what methods they are most comfortable with and how they see themselves being best able to contribute.

Several research methods have previously been described for involving individuals with aphasia in person-centered activities, like interviews for research purposes. Interviews provide researchers with rich, detailed, qualitative data for understanding the experiences of participants and the meaning they make from their experiences [13]. However, interviewing patients with aphasia can be difficult and therefore alternative methods are often required. The use of video as a visual means of data collection [7] has been shown to be an effective method for qualitative research in health care as a means of reflection and elicitation. While Iedema et al [7] described the advantages of reviewing the video footage from a health care provider perspective, this experience was concerned with the processes and procedures within the workplace. Video ethnography has been shown to be successful for analyzing consulting methods, for remodeling within a general practice setting [8], and to help staff to reflect on their work [14]. It was also noted that the simple presence of the video camera was regarded as having a positive influence by eliciting a more reflexive work method.

Dalemans et al [9] suggested several adjustments to qualitative research techniques when working with patients with aphasia, including reducing the cognitive load by reducing the content, utilizing clear visual structure when able, and utilizing alternative forms of communication, such as pictures, writing, gestures, and mime. These methods of communication and interaction with patients with aphasia in an interview situation may provide more open and relaxed communication between the interviewer and the interviewee. These adjustments may also be in line with the rehabilitation methods utilized poststroke and might be considered familiar and less intrusive than methods such as video. Luck and Rose [15] expressed similar findings, suggesting that researchers need to “step out of their traditional role” by changing the way they ask questions, offer ideas to patients, and use various supportive conversation techniques.

Including participants with aphasia in qualitative research into stroke service redesign is important for ensuring that the provision of health care services meets the needs of all patients. The experiences of patients with aphasia in the hospital and in the community provide valuable information toward ensuring that all patients are afforded the services that they require. These participants identified that it is important to deliver assessment and communication tools and rehabilitation services that meet the specific needs of different patients with aphasia, as well as other post-stroke complications. Excluding patients with aphasia from this research would have reduced the potential sample size by about one-quarter and resulted in a huge gap in the experiential data. The focus of the interview data was not about the patients’ communication deficits, although it was expected and confirmed in some cases that communication difficulties had hindered, or at least altered, their experiences in the hospital. Without including this feedback in the co-redesign process, gaps in service provision for aphasic patients could remain.

Utilizing various methods of communication is often necessary to ensure a full and information-rich experience for both the interviewer and the interviewee, and documenting how the researcher does this and how this experience changes the interview and data collected is an important component of the inclusive, co-design methodology.

### Limitations

The major limitations of this study were the small cohort of patients and the lack of information collected before the interviews about their potential communication deficits, which had a profound effect on the experience of the researchers conducting the face-to-face interviews. Without a full understanding of the needs of the participant, the research team was ill-prepared to collect useful data, which also created angst among them. Improved screening processes beforehand would have been beneficial to aid in the design of the interview and data collection methods. In the future, specific strategies, including the presence of more than one interviewer or video recording rather than audio data collection, may be useful for qualitative research participants with aphasia or other communication deficits. Research investigating the views of individuals with aphasia on the best methods for interviews would be an important step in developing patient engagement strategies for health service improvement.

### Conclusion

The reliance upon the written word in the form of transcriptions of audio-recorded interviews in qualitative research reinforces the limitations of using just this type of communication. For future qualitative research that intends to engage individuals with aphasia, researchers must devise appropriate strategies and methods beforehand to maximize the contributions and valuable communications of these participants, as well as reduce the possibility for negative emotional responses in ill-prepared researchers.
